# Adipose-derived exosomes block muscular stem cell proliferation in aged mouse by delivering miRNA *Let-7d-3p* that targets transcription factor HMGA2

**DOI:** 10.1016/j.jbc.2022.102098

**Published:** 2022-06-06

**Authors:** Maki Itokazu, Yuta Onodera, Tatsufumi Mori, Shinji Inoue, Kotaro Yamagishi, Akihiro Moritake, Natsumi Iwawaki, Kanae Shigi, Toshiyuki Takehara, Yuji Higashimoto, Masao Akagi, Takeshi Teramura

**Affiliations:** 1 Department of Rehabilitation Medicine, Kindai University Faculty of Medicine, Osaka-Sayama, Osaka, Japan; 2 Institute of Advanced Clinical Medicine, Kindai University Hospital, Osaka-Sayama, Osaka, Japan; 3 Life Science Institute, Kindai University, Osaka-Sayama, Osaka, Japan; 4 Department of Orthopedic Surgery, Kindai University Faculty of Medicine, Osaka-Sayama, Osaka, Japan

**Keywords:** perimuscular adipose tissue, muscular satellite cells, stem cell aging, interorgan communication, Let-7, HMGA2, AdMSC, adipose-derived mesenchymal stem cell, A-PMAT, aged PMAT, DMEM, Dulbecco’s Modified Eagle Medium, FACS, fluorescence activated cell sorting, FCS, fetal calf serum, IL-1β, interleukin 1β, MPC, muscular progenitor cell, MSC, mesenchymal stem cell, NF-κB, nuclear factor-kappa B, PMAT, perimuscular adipose tissue, qRT-PCR, quantitative reverse-transcription polymerase chain reaction, TNF-α, tumor necrosis factor α, Y-PMAT, young PMAT

## Abstract

Sarcopenia is an aging-associated attenuation of muscular volume and strength and is the major cause of frailty and falls in elderly individuals. The number of individuals with sarcopenia is rapidly increasing worldwide; however, little is known about the underlying mechanisms of the disease. Sarcopenia often copresents with obesity, and some patients with sarcopenia exhibit accumulation of peri-organ or intra-organ adipose tissue as ectopic fat deposition, including atrophied skeletal muscle. In this study, we showed that transplantation of the perimuscular adipose tissue (PMAT) to the hindlimb thigh muscles of young mice decreased the number of integrin α7/CD29-double positive muscular stem/progenitor cells and that the reaction was mediated by PMAT-derived exosomes. We also found that the inhibition of cell proliferation was induced by *Let-7d-3p* miRNA that targets HMGA2, which is an important transcription factor for stem cell self-renewal, in muscular stem/progenitor cells and the composite molecular reaction in aged adipocytes. Reduction of Let-7 miRNA repressor Lin28 A/B and activation of nuclear factor-kappa B signaling can lead to the accumulation of *Let-7d-3p* in the exosomes of aged PMAT. These findings suggest a novel crosstalk between adipose tissue and skeletal muscle in the development of aging-associated muscular atrophy and indicate that adipose tissue–derived miRNAs may play a key role in sarcopenia.

Sarcopenia, which is characterized by the loss of skeletal muscle mass and strength in older individuals, is drawing attention as a major cause of decreased quality of life. It is known that low-grade inflammation occurs chronically in aged tissue and is thought to be a major contributor to the decline in the regenerative capacity of muscles (1, 2). However, the molecules that mediate the onset or progression of sarcopenia in aged muscles are not well understood.

Several studies suggest that disruption of the relationship between adipose tissue and muscle tissue may be involved in the onset and progression of sarcopenia (3, 4, 5). Recent studies have shown that adipose tissue surrounding skeletal muscle, known as perimuscular adipose tissue (PMAT), increases in parallel with muscle atrophy, along with decreased type II myofiber cell size (6, 7).

Adipose tissue plays important roles not only in regulating whole-body energy metabolism through its storage function but also in the production of various secreted factors called adipocytokines, such as leptin and adiponectin. Imbalance of the adipose-secreted factors, *i.e*., overproduction of proinflammatory adipocytokines and diminished expression of anti-inflammatory cytokines in obesity, is considered to be responsible for systemic metabolic dysfunction, cardiovascular disease (8), and regeneration inhibition in diverse tissues (9).

In addition to adipocytokines, exosomes are important mediators of intercellular and intraorgan communication systems. Exosomes carry various molecules, including proteins, DNA, mRNA, and microRNAs (miRNAs), and regulate normal biological functions, such as tissue regeneration and development. In addition, exosome-mediated communication has been reported to be important (10, 11, 12). Among the molecules contained in exosomes, the biological response of miRNAs has been shown to play an important role in tissue development, regeneration, homeostasis, and disease pathogenesis. So far, it has been demonstrated that some aging-associated changes in tissue integrity are controlled by miRNAs.

In this study, we hypothesized that the age-related decrease in the number of muscular progenitor/stem cells is caused by a disruption of interorgan communication between muscle and adipose tissues and that cell proliferation and/or self-renewal are inhibited by molecules secreted from PMAT from aged mice (aged PMAT; A-PMAT). Here, we showed that exosomes isolated from A-PMAT contain high levels of *Let-7d-3p* miRNA and inhibit cell proliferation by suppressing HMGA2, a transcription factor required for stem cell proliferation.

## Results

### Transplantation of PMAT derived from aged mice reduced the number of MPCs ***in young mice***

**Figure 1:**
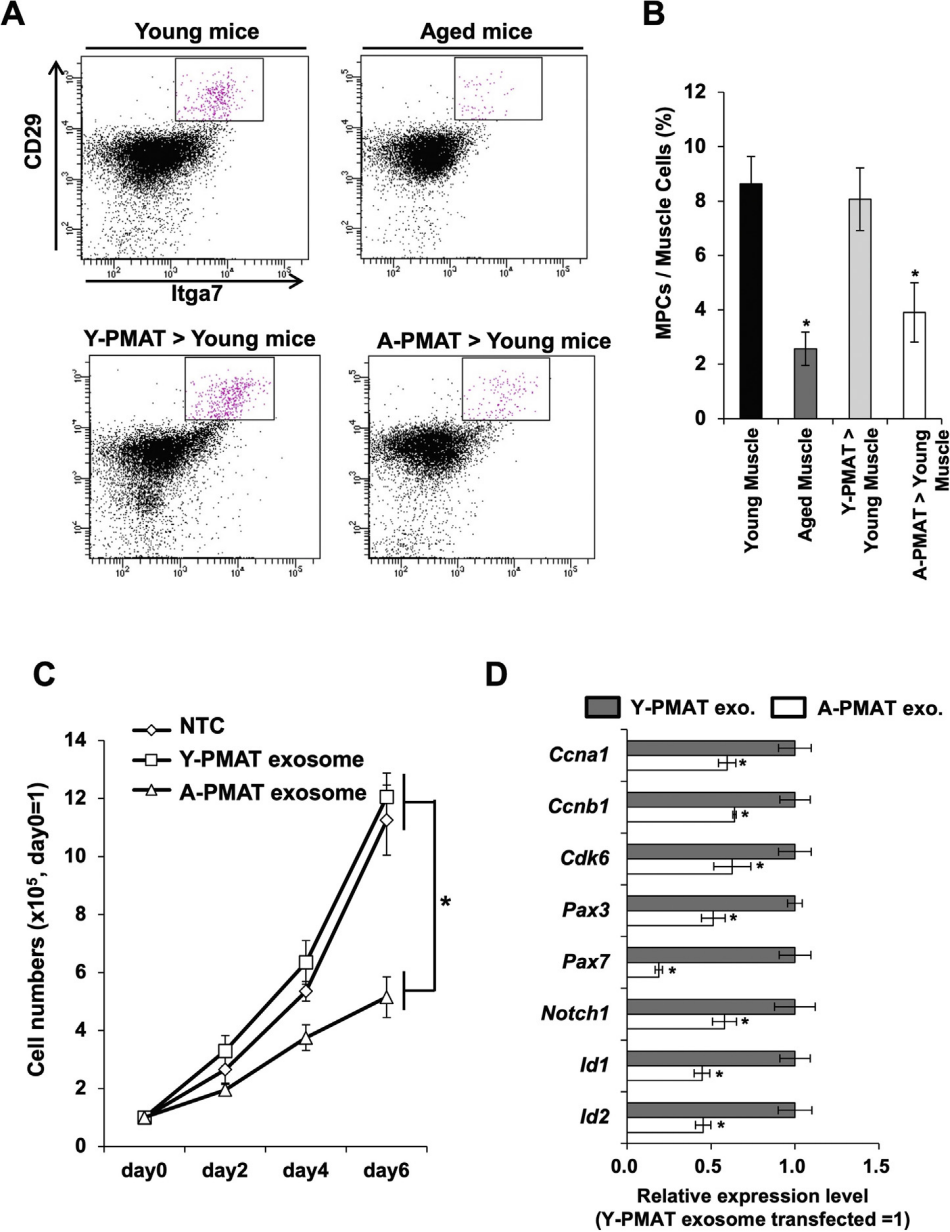
**Transplantation of PMAT decreased the number of MSCs**. *A*, gating of MPCs including muscular satellite cells as CD29/Itga7^positive^ and CD11b/CD31/CD45/Ter119^negative^. *B*, cell numbers of CD29/Itga7^positive^ MPCs in muscles transplanted with PMAT derived from aged or young mice. *C*, cell proliferation of the primary culture of mouse myoblast cells treated with exosomes isolated from Y-PMAT (young) or A-PMAT (aged). *Asterisk* indicates a significant difference at *p* <0.05. NTC, no-treatment control. *D*, expression of cell cycle- and stemness-associated genes in primary myoblast cells treated with exosomes derived from Y-PMAT (young) or A-PMAT (aged). *Asterisk* indicates a significant difference at *p* <0.05. A-PMAT, aged PMAT; MSC, mesenchymal stem cell; PMAT, perimuscular adipose tissue; Y-PMAT, young PMAT.

Muscular progenitor cell fraction including satellite cells in muscular tissues, referred to as “MPCs” in this study, was identified as CD29/ITGA7 double-positive cells among cells of nonhematopoietic lineage, which were discriminated as CD11b/CD31/CD45/Ter119 negative. In the muscles of 3-week-old mice, the average proportion of MPCs was approximately 9%. On the contrary, the number of MPCs decreased to approximately 3% in 2-years aged mice. The CD29/ITGA7 double-positive MPC population in young mice expressed cell proliferation–associated genes and stemness-related genes at higher levels compared with that in aged mice (Fig. S1). To examine whether PMAT induced a decrease in the number of MPCs in aged mice, we performed tissue transplantation between young and aged mice. Intragroup transplantation, *i.e.,* the transplantation of PMAT from young mice into young mice, did not affect the number of MPCs. In contrast, transplantation of PMAT from aged mice into young mice significantly reduced the number of MPC fractions to 50%, compared with that in the nontreated control (Fig. 1, *A* and *B*).

### Exosomes secreted from A-PMAT affected cell proliferation of mice primary MPCs

To examine whether exosomes mediated the A-PMAT-induced reduction of MPCs in A-PMAT-transplanted mice, we isolated exosomes from A-PMAT and added them to the primary culture of myoblast cells, which contained CD29/ITGA7 double-positive MPCs at approximately 84%. Among the MPCs, Pax7-positive stem cells constituted approximately 34% of the cell population in the muscles of young mice (Fig. S2). Significant changes were not observed in myoblast cells treated with exosomes derived from the PMAT of young mice (young PMAT; Y-PMAT). In contrast, treatment with exosomes derived from A-PMAT dramatically suppressed cell proliferation (Fig. 1*C*). In myoblast cells treated with A-PMAT exosomes, the expression of cell cycle–associated molecules (such as *Ccna1*, *Ccnb1*, and *Cdk6*) and stemness-related genes (such as *Pax3*, *Pax*7, *Notch1*, *Id1,* and *Id2*) were suppressed (Fig. 1*D*).

### Let-7d-3p was highly expressed in A-PMAT exosomes and inhibited cell proliferation of the primary MPCs

**Figure 2:**
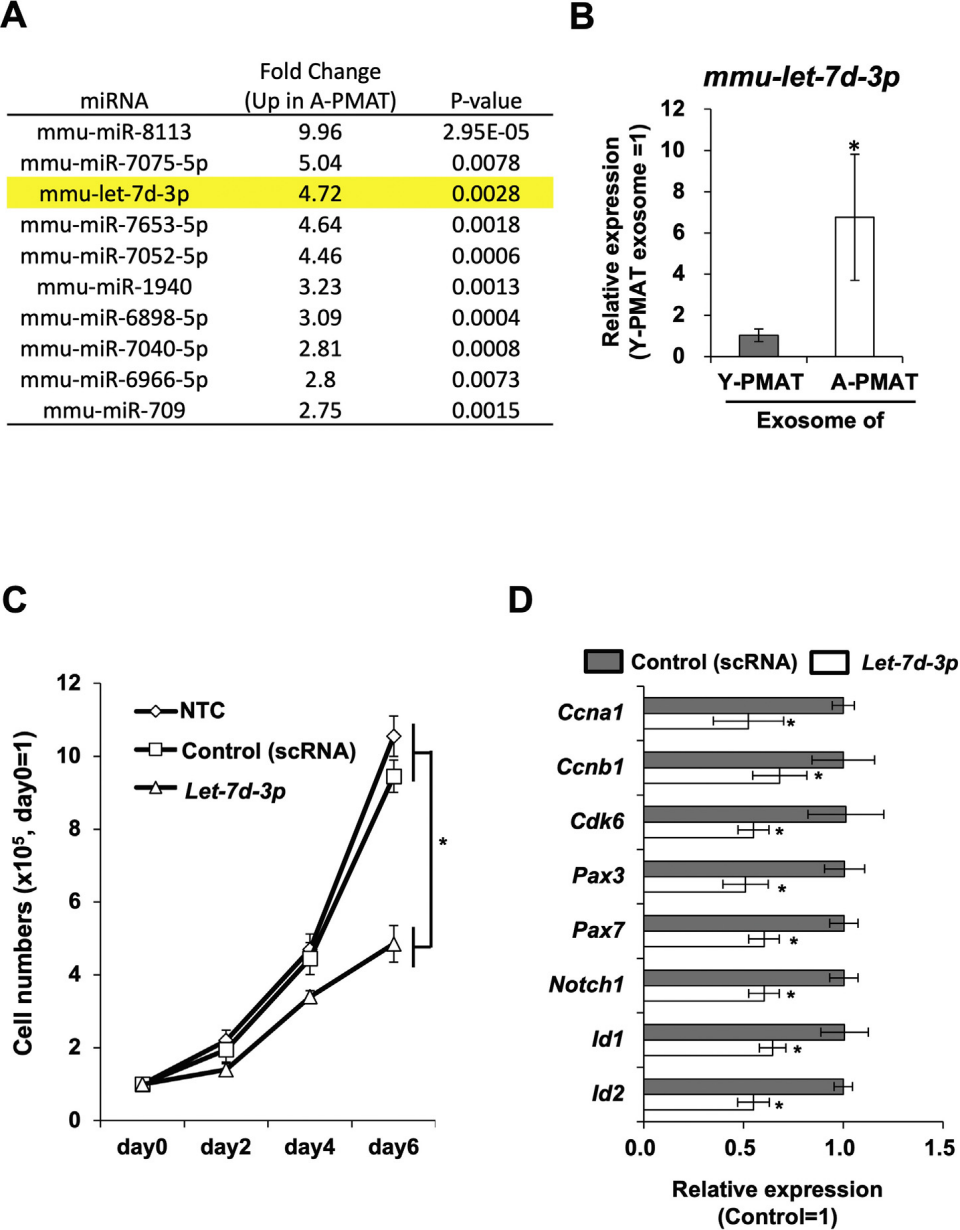
***Let-7d-3p* was highly expressed in A-PMAT-derived exosomes and inhibited cell proliferation of MPCs through suppression of HMGA2**. *A*, List of top 10 miRNA highly expressed in exosomes derived from A-PMAT. *B*, expression level of *Let-7d-3p* in exosomes from Y-PMAT (young) or A-PMAT (aged). *C*, growth curve of primary myoblast cells treated with A-PMAT–derived exosomes. *Asterisk i*ndicates a significant difference at *p* <0.05. *D*, expression of cell cycle- and stemness-associated genes in primary myoblast cells treated with scrambled RNA or *mmu-Let-7d-3p* miRNA mimic. Control cells were treated with scrambled RNA. *Asterisk* indicates a significant difference at *p* <0.05. A-PMAT, aged PMAT; PMAT, perimuscular adipose tissue; Y-PMAT, young PMAT.

To identify the molecule responsible for the exosome-mediated inhibition of cell proliferation, we compared miRNA expression between exosomes isolated from A-PMAT and Y-PMAT. In this experiment, we found that 28 miRNAs were upregulated by more than 2-fold with significant difference (*p* < 0.05) in A-PMAT (Table S1). For detailed examination, we narrowed down the list to 15 miRNAs with a high expression score (<5) and a low *p*-value (*p* < 0.01). Among the top 10 miRNAs with the highest fold change (Fig. 2*A*), we selected *Let-7d-3p* because only *Let-7d-3p* was conserved between humans and mice. Quantitative reverse-transcription polymerase chain reaction (qRT-PCR) of concentrated exosomes from six young or six aged mice revealed that *Let-7d-3p* expression level was significantly increased in A-PMAT (Fig. 2*B*). We then investigated the effect of *Let-7d-3p* on the proliferation of MPCs using primary myoblast cells isolated from young mice. As expected, transfection of *Let-7d-3p* mimic RNA inhibited cell proliferation (Fig. 2*C*). qRT-PCR analysis showed that the expression of cell proliferation–associated genes and stemness-related genes was suppressed by *Let-7d-3p* miRNA mimic transfection (Fig. 2*D*).

### Suppression of HMGA2 resulted in the A-PMAT exosome/Let-7d-3p-induced reduction of primary myoblast cell proliferation

**Figure 3:**
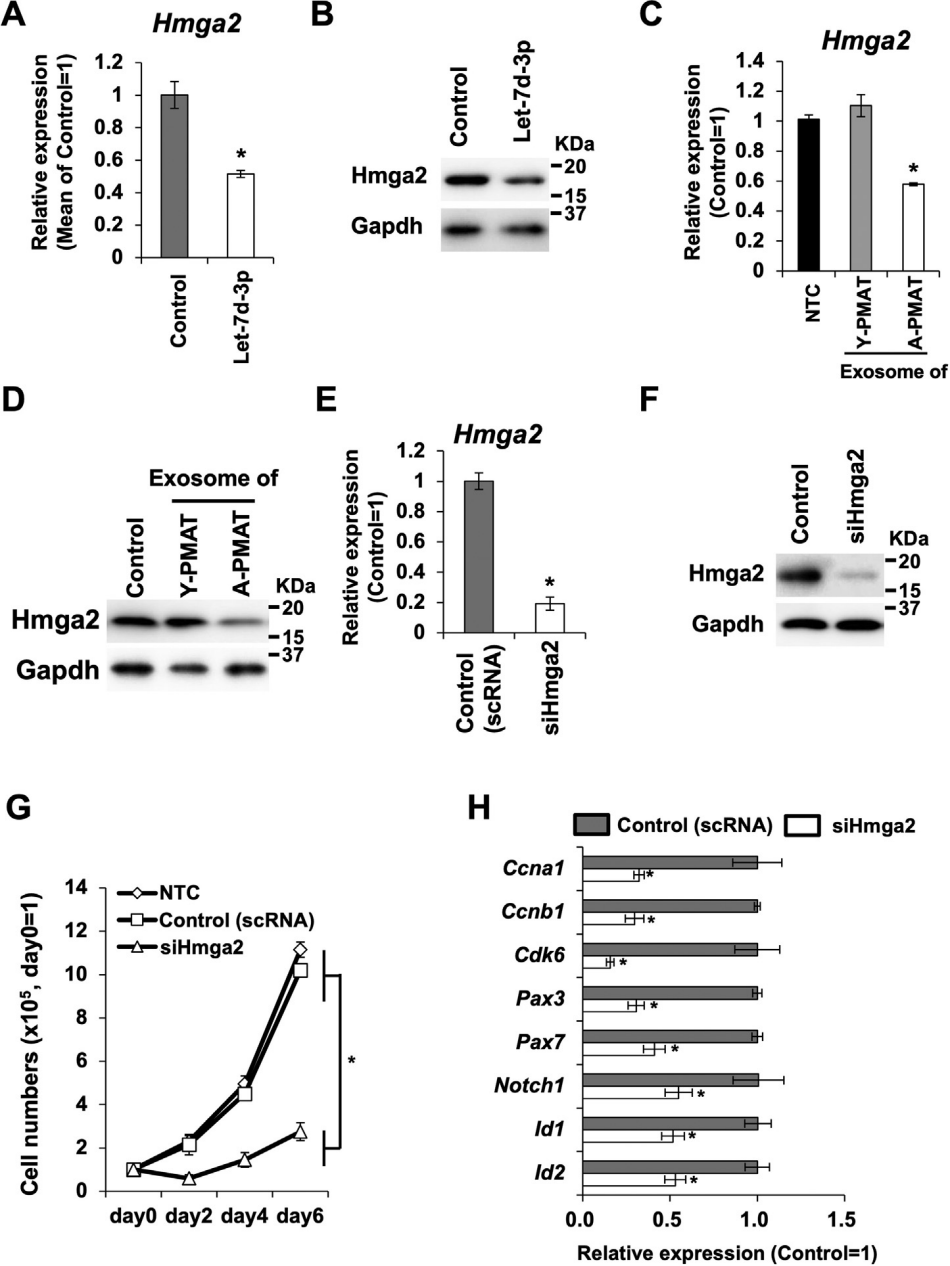
**Suppression of HMGA2 was a direct reason for *Let-7d-3p–*induced reduction of cell proliferation and alteration in gene expression**. *A*, expression change in *Hmga2* RNA due to *Let-7d-3p* mimic RNA transfection in mouse primary MPCs. *Asterisk* indicates a significant difference at *p* <0.05. Control, MPCs transfected with scrambled RNA. *B*, Western blotting analysis for the expression of Hmga2 protein in *Let-7d-3p* mimic RNA-transfected mouse primary MPCs. Control, MPCs transfected with scrambled RNA. *C*, expression of *Hmga2* RNA in primary MPCs treated with exosomes derived from Y-PMAT (young) or A-PMAT (aged). *Asterisk* indicates a significant difference at *p* <0.05. *D*, expression of Hmga2 protein in primary MPCs treated with exosomes derived from Y-PMAT (young) or A-PMAT (aged). *E*, expression of *Hmga2* RNA in primary MPCs treated with siRNA against *Hmga2* (siHmga2). Control cells were treated with scrambled RNA (scRNA). Control, MPCs treated with scrambled RNA. *Asterisk* indicates a significant difference at *p* <0.05. *F*, expression of Hmga2 protein in primary MPCs treated with siRNA against *Hmga2* (siHmga2). Control, MPCs treated with scrambled RNA. *G*, growth curve of primary MPCs treated with siHmga2 or scrambled RNA (control). *Asterisk* indicates a significant difference at *p* <0.05. *H*, expression of cell cycle- and stemness-associated genes in primary MPCs treated with siHmga2 or scrambled RNA (control). *Asterisk* indicates a significant difference at *p* <0.05. A-PMAT, aged PMAT; MSC, mesenchymal stem cell; PMAT, perimuscular adipose tissue; Y-PMAT, young PMAT.

Considering the results of target prediction using TargetScan and miRDB (Table S2), as well as the previous evidence, we hypothesized that the targeting of *Hmga2* by *Let-7d-3p* resulted in the reduction of proliferation in the primary culture of myoblast cells. As expected, the introduction of *Let-7d-3p* resulted in 50% downregulation of *HMGA2* expression in mouse primary myoblast cells (Fig. 3*A*). Western blotting analysis also showed HMGA2 downregulation following *Let-7d-3p* transfection (Fig. 3*B*). Importantly, treatment with purified exosomes from A-PMAT also reduced HMGA2 expression in mouse primary myoblast cells (Fig. 3, *C* and *D*). Consistent with our hypothesis, suppression of HMGA2 by siRNA treatment led to decreased cell number (Fig. 3*G*), downregulation of cell cycle, and decrease in stemness-associated gene expression (Fig. 3*H*) in primary myoblast cells containing stem cells. On the contrary, HMGA2-overexpressing myoblast cells did not show the suppression of cell proliferation or a decrease in stemness-associated gene expression following the transfection of *Let-7d-3p* mimic (Fig. S4).

### Activation of NF-κB by inflammatory cytokines and attenuated expression of Lin28A/B led to the upregulation of Let-7d-3p in adipocytes

We hypothesized that aging-associated inflammation, which is frequently observed in aged adipose tissue, upregulates *Let-7d-3p* in A-PMAT.

**Figure 4:**
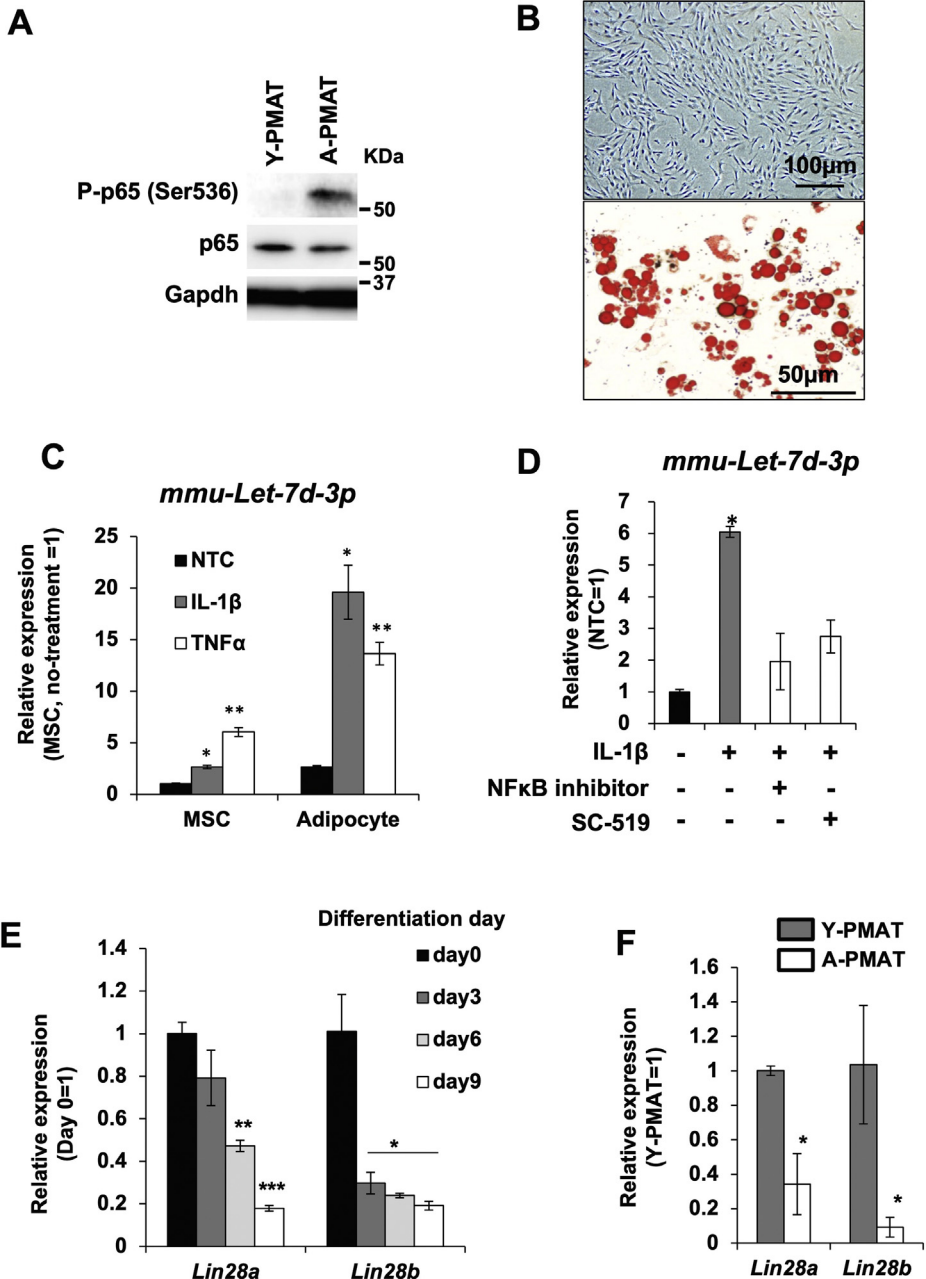
**Role of NF-κB and proinflammatory cytokines in the expression of *Let-7d-3p* in adipocytes induced from AdMSCs**. *A*, phosphorylation status of the serine536 (S536) residue on p65 in Y-PMAT (young) and A-PMAT (aged). *B*, adipose-derived MSCs (AdMSCs) and adipocyte induced from AdMSCs. Lipid droplets accumulated in adipose cells were visualized by Oil-Red-O staining. *C*, effect of proinflammatory cytokines IL-1β and TNF-α on the expression of *mmu-Let-7d-3p* in adipocytes induced *in vitro* from AdMSCs. *D*, inhibition of NF-κB activity blocked IL-1β–induced upregulation of *Let-7d-3p*. *Asterisk* indicates a significant difference at *p* <0.05. *E*, time-dependent decrease in *Lin28a* and *Lin28b* expression during adipocyte differentiation. *Asterisk* indicates a significant difference at *p* <0.05. *F*, expression of *Lin28A* and *Lin28B* in Y-PMAT (young) and A-PMAT (aged). A-PMAT, aged PMAT; PMAT, perimuscular adipose tissue; Y-PMAT, young PMAT.

To confirm that inflammatory reactions result in the upregulation of *Let-7d-3p*, we observed the activity of nuclear factor-kappa B (NF-κB), which is an important mediator of inflammatory reactions. In A-PMAT, the Ser^536^ residue of p65, which is an essential subunit of the NF-κB complex, was dramatically phosphorylated (Fig. 4*A*). We then treated mouse adipocytes (Fig. 4*B*) produced *in vitro* from adipose-derived mesenchymal stem cells (AdMSCs) with interleukin 1β (IL-1β) and tumor necrosis factor α (TNF-α) and observed expression of *Let-7d-3p* by qRT-PCR. In both the adipocytes treated with IL-1β and TNF-α, *Let-7d-3p* expression was significantly increased (Fig. 4*C*). Furthermore, the IL-1β-induced upregulation of *Let-7d-3p* was blocked by treatment with the NF-κB inhibitor or ATP-competitive IKKβ inhibitor SC-514 (Fig. 4*D*). Interestingly, the Let-7d-3p expression response to the proinflammatory cytokines was very weak in undifferentiated AdMSCs (Fig. 4*C*). To clarify why the reactivity of *Let-7d-3p* expression to proinflammatory cytokines was different between undifferentiated AdMSCs and adipocytes, we assessed the expression of *Lin28A/B*, which is a bipartite RNA-binding protein that posttranscriptionally inhibits *Let-7* miRNA maturation and function. Quantitative RT-PCR analysis showed that the expression of *Lin28A* and *Lin28B* was decreased by adipocyte differentiation (Fig. 4*E*). Importantly, expression levels of *Lin28A* and *Lin28B* were lower in A-PMAT than in Y-PMAT (Fig. 4*F*).

### Upregulation of *Let-7d-3p* was observed in adipose tissue of aged donors

**Figure 5:**
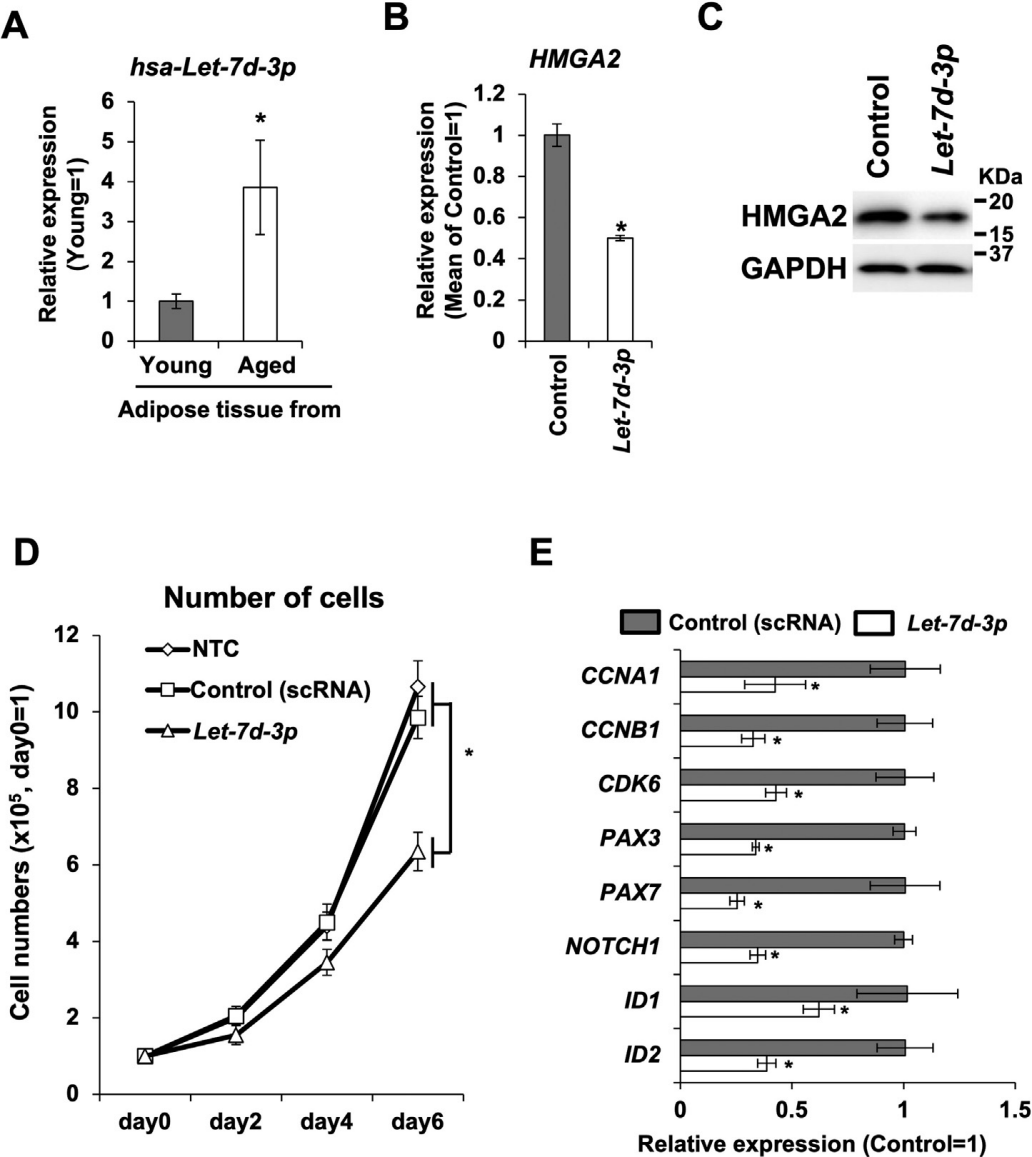
**Expression of *Let-7d-3p* and its function in human primary MPCs**. *A*, expression of *hsa-Let-7d-3p* in adipose tissues from young and aged donors. *Asterisk* indicates a significant difference at *p* <0.05. *B*, expression of *HMGA2* RNA in human primary MPCs transfected with *Let-7d-3p* mimic RNA. *Asterisk* indicates a significant difference at *p* <0.05. *C*, expression of HMGA2 protein in human primary MPCs transfected with *Let-7d-3p* mimic RNA. *D*, growth curve of human primary MPCs treated with *Let-7d-3p* mimic RNA or scrambled RNA (control). *Asterisk* indicates a significant difference at *p* <0.05. *E*, expression of cell cycle and stemness-associated genes in human primary MPCs treated with *Let-7d-3p* mimic RNA or scrambled RNA (control). *Asterisk* indicates a significant difference at *p* <0.05.

To confirm whether the aging-associated upregulation of *Let-7d-3p* also occurs in human adipose tissue, we prepared total RNA from the adipose tissue of young and aged donors and performed qRT-PCR. Consistent with the results in mouse tissues, *Let-7d-3p* was highly expressed in the adipose tissue of aged donors (Fig. 5*A*). To determine whether *Let-7d-3p* expression blocks cell proliferation in human myoblast cells, we transfected *Let-7d-3p* miRNA mimics into the primary myoblast cells of young donors. In human cells, *Let-7d-3p* suppressed HMGA2 expression (Fig. 5, *B* and *C*). Furthermore, reduction of cell proliferation and decreased expression of cell cycle-associated and stemness-associated genes were observed in *Let-7d-3p-*transfected human primary myoblast cells (Fig. 5, *D* and *E*).

## Discussion

Adipose tissues and muscles communicate closely through secretion and receipt of various cytokines, myokines, and metabolites (13, 14, 15).

We showed herein that the transplantation of A-PMAT decreased the number of muscle progenitors, including satellite cells, and exosomes derived from A-PMAT suppressed the proliferation of muscular progenitor/stem cells.

Exosomes released by adipose tissue are involved in the physiological regulation of various tissues, such as immune homeostasis (16, 17) or angiogenesis (18). Pathophysiologically, adipose-derived exosomes from obese individuals accelerate the formation of macrophage foam cells, promote inflammation, increase the risk of atherosclerosis (19), and increase the risk of macrophage-induced insulin resistance (20).

The exosome components exhibit different profiles depending on the age and/or disease conditions of the donor tissues (21). In particular, exosomes contain various miRNAs that vary depending on tissue, diseases, and donor age. To identify the miRNA responsible for the inhibition of cell proliferation, we performed miRNA array analysis with Y-PMAT- and A-PMAT-derived exosomes and found that the miRNA *Let-7d-3p* was upregulated in exosomes derived from A-PMAT.

*Let-7* family members are direct and strong regulators of mitogenic genes, such as the RAS family genes. *K-RAS*, *N-RAS*, *H-RAS*, and *MYC* mRNAs contain *Let-7* binding sites in the 3′UTR sequences (22). In normal cells, HMGA2 has been reported as a target of *Let-7d* (23, 24, 25), and this mechanism changes the cellular phenotype (26). The DNA-binding protein HMGA2 is abundantly expressed in fetal and young somatic stem cells, such as hematopoietic stem cells or neural stem cells, and contributes to adult stem cell stemness by repressing the expression of p16^INK4A^ and p19^ARF^ (27). Moreover, in myoblast cells, HMGA2 is involved in proliferation and differentiation regulation (28, 29). Consistent with the findings of previous studies, transfection of *Let7d-3p* strongly suppressed cell proliferation in myoblast cells, and HMGA2 expression was decreased. Decreased expression of HMGA2 was also observed in aged MPCs and MPCs from the model mice transplanted with A-PMAT. Furthermore, treatment with concentrated exosomes derived from A-PMAT resulted in the suppression of HMGA2 expression. On the contrary, transfection of siRNA against *HMGA2* significantly inhibited the proliferation of myoblast cells and the expression of cell cycle–related genes, indicating that the central mechanism for the inhibition of myoblast cell proliferation caused by exosomes secreted from adipose tissue in aging mice involves the targeting of *HMGA2* by *Let-7d* miRNA.

The addition of A-PMAT-derived exosomes or transfection with *Let-7d-3p* also altered the expression of genes related to differentiation (Fig. S6). Unfortunately, it is difficult to determine which genes were targeted to cause changes in the expression of genes related to muscle differentiation. The role of HMGA2 in sustaining the self-renewal of adult stem cells possibly by blocking their differentiation has been described in neural stem cells and mesenchymal stem cells (30, 31). Moreover, Let-7d has been reported to promote neural differentiation by suppressing TLX in neural stem cells (32). To elucidate the exact function of Let7d in stem cell regulation, it is necessary to deeply study its relationship with molecules involved in undifferentiated regulation.

Next, we examined the mechanism of how *Let7d-3p* was upregulated in A-PMAT.

The expression levels of proinflammatory cytokines, such as IL-1β, IL-6, and TNF-α, showed major differences between young and aged adipose tissues. These inflammatory cytokines activate MAP kinases and induce various reactions in aged tissues. NF-κB is a central transcription factor that regulates inflammatory responses (33). Garzon *et al*. reported that NF-κB activates Let7 during the granulocytic differentiation of NB4 cells induced by all-trans-retinoic acid (34). Wang *et al*. also revealed that the Let-7 promoter is highly responsive to NF-kB, and ectopic expression of subunits of NF-kB (p65/RelA) significantly activates Let-7 promoter. In patients with multiple sclerosis, a high-level expression of Let7d and positive correlation with IL-1b expression were shown (35).

To confirm whether the inflammatory response was activated by A-PMAT, we first observed the activation of NF-κB, a central molecule in the inflammatory response. The p65 subunit, the regulatory center of NF-κB activity, was clearly phosphorylated at p65 serine 536 (S536) in A-PMAT. The S536 residue of the p65 subunit represents the site with the most potent inducible phosphorylation in response to inflammatory stimuli and is thought to regulate NF-κB activity (36). In Y-PMAT, no phosphorylation of p65-S536 was detected. Next, to confirm that the expression of *Let-7d-3p* is activated by the NF-κB pathway, we produced adipocytes *in vitro* from AdMSCs and stimulated them with IL-1β or TNF-α in adipose tissues. In adipocytes, both IL-1β and TNF-α activated the expression of *Let-7d-3p*. This result is consistent with a previous evidence that the promoter activity of Let7 is activated by TNF-α (37). In contrast, the addition of an NF-κB activation inhibitor quenched the effect of IL-1β. This experiment revealed that *Let-7d-3p* expression was induced by the activation of the NF-κB pathway by proinflammatory cytokines. Interestingly, the proinflammatory cytokine-induced *Let-7d-3p* expression response was very weak in undifferentiated AdMSCs. We hypothesized that the miRNA processing inhibitor protein Lin28, as a molecule regulating the cell-type or context dependent reaction, was involved. Mammalian Lin28 exists as two highly conserved paralogues, Lin28a and Lin28b, both of which repress Let-7 expression (38, 39). Lin28 regulates *Let-7* by binding to pre-Let-7 in a region called the precursor element, located in the hairpin loop (40). Regarding the associations of the inflammatory molecules Lin28 and Let7, Lin28 is activated by NF-κB, whereas Let7a expression is repressed in normal immortalized mammary epithelial cell line MCF10A (41). In previous studies, a differentiation-dependent decrease in Lin28 expression was reported in osteogenesis from AdMSCs (42, 43). Consistently, we found that both *Lin28a* and *Lin28b* expression was significantly suppressed by adipocyte differentiation. Furthermore, decreased expression of Lin28a and Lin28b was observed in A-PMAT. This result is consistent with the finding that Let-7d-3p expression is upregulated in A-PMAT. Lin28 expression is mainly restricted to undifferentiated stem cell types, such as embryonic stem cells, certain transformed cell lines, and adult stem cells, including adipocyte stem cells (44). Thus, Lin28 expression in stem cells may occur as a mechanism to prevent cytokine-stimulated activation of *Let-7d* and the subsequent proliferation inhibition.

From these notions, we concluded that Let7 expression is regulated *via* at least two mechanisms (1): *via* transcriptional regulation by transcription factors including p65 and (2) *via* posttranscriptional regulation by Lin28. This dual regulatory mechanism may be responsible for the tissue or cell-type dependent reaction of Let7 expression to the inflammatory molecules. To date, numerous studies have reported that cellular characteristics, gene expression, differentiation potentials, and cell numbers of AdMSCs in adipose tissues are altered by aging (45, 46, 47). Although it remains to be determined which cell type in PMAT expresses Lin28, the abnormal differentiation and deterioration of AdMSCs by aging may lead to decreased Lin28 expression, creating an environment that is prone to inflammation-induced upregulation of *Let-7d*.

As an important finding, the overexpression of *Let-7d-3p* by aging, targeting of HMGA2 by *Let-7d-3p*, and *Let-7d-3p*-induced reduction of cell proliferation were reproducible in the human adipose tissues and myoblast cells. These results showed that interorgan communication supported by the *Let-7d*-HMGA2 axis is conserved across species, suggesting that it is responsible for age-related tissue stem cell loss or impaired tissue regeneration.

The present study showed that cell pools of muscular stem/progenitor cells affected interorgan communication between adipocytes and muscles and the exosomes secreted from PMAT decreased the cell numbers in aged mice. We also showed that the exosomes of aged mice contained *Let-7d-3p* miRNA, which was upregulated by attenuation of Lin28 in adipocytes, and it reduced the proliferation of muscular stem/progenitor cells through suppression of HMGA2. Although it is clear that epidemiological evaluation is required for patients with aging, obesity, or both, our results reveal an important mechanism underlying intraorgan communication-based muscular atrophy due to aging.

## Experimental procedures

### Ethics statement

The Institutional Review Board of the Kindai University Faculty of Medicine approved the human tissue procurement protocol. The tissue specimens used in this study were collected at the margin of devitalized and healthy-appearing tissue that would otherwise be discarded as surgical waste. Tissue samples were retrieved from young patients (4 males and 2 females, mean age = 31.8 years) and aged patients (4 males and 8 females, mean age = 77.5) that received total hip arthroplasty and anterior cruciate ligament reconstruction at the Kindai University Hospital.

All animal handling, care, operation, and sacrifice procedures were approved by the Institutional Animal Care and Use Committee at Kindai University and were performed in accordance with institutional guidelines and regulations.

### Transplantation of PMAT

Five-week-old (young) male and 2-year-old (aged) male C57BL/6 mice were used as model animals. Adipose tissue was collected from the thigh and popliteal regions of young or aged mice, washed with phosphate-buffered saline (PBS), cut into 3-mm squares with a scalpel, and divided into 100 mg each. For transplantation, recipient mice were anesthetized with isoflurane, and a 1-cm incision was made on the ventral side of the thigh. Next, a 6- to 8-mm pocket was created under the dorsal fascia using a fine tweezer without damaging the muscle tissue. Adipose tissue treated as described above was transplanted into the slit. Sampling was performed at 72 h after surgery.

### Isolation of exosomes from PMAT

PMAT of young and aged mice was minced and cultured overnight in Dulbecco’s Modified Eagle Medium (DMEM; Thermo Fisher Scientific) at 37 °C under 5% CO_2_. The supernatant was centrifuged at 3000*g* for 15 min at 4 °C to remove debris, and the remaining supernatant was concentrated to 1 ml using an Amicon Ultra-15 (Merck Millipore) *via* centrifugation in a swing-out rotor at 4 °C and 4000*g*. The resulting exosome-enriched medium was resuspended in 1 ml of PBS and subjected to high-speed centrifugation at 100,000*g* on a 30% sucrose cushion at 4 °C for 2 h. The resulting exosome-enriched pellet was resuspended in 50 μl of 1 × PBS and used for further analysis. Collected exosomes were confirmed by Western blotting analysis and electron microscopy. The concentrated exosomes were normalized per wet tissue weight and used as a material. Exosomes from 500 mg of adipose tissue were added per 1 × 10^5^ cells in the exosome treatment experiment.

### Fluorescence activated cell sorting analysis of mouse muscle stem/progenitor cells

An overdose of isoflurane was administered to 5-week-old C57BL/6J male mice. The euthanized mice were deblooded by refluxing with saline. After sterilization with iodine-based disinfectant and 70% ethanol, the thigh muscles were aseptically removed and washed three times with PBS. Epidermal tissues, nerves, and ligaments were carefully removed using microdissection scissors. The muscles were minced using a scalpel and digested in DMEM containing 0.1% collagenase (type II; Worthington) and 5% fetal calf serum (FCS, Corning) for 30 min. After careful pipetting, the sample was filtered using a 100-, 70-, and 40-μm cell strainer (Greiner Japan). The digested cells were washed three times with 10% FCS-DMEM, mounted on 20% Percoll (GE Healthcare Bio-Sciences AB) and centrifuged at 3000 rpm for 20 min. After centrifugation, the defined interphase was collected, washed with 10% FCS-DMEM, and centrifuged again at 1500 rpm for 10 min. The resulting cell pellets were resuspended in 1%FCS-DMEM supplemented with Hepes (Wako Pure Chemical Corp) and incubated with APC-conjugated anti-CD29 (HMβ1-1; BioLegend), PE-conjugated anti-ITGA7 (3C12; MBL), FITC-conjugated anti-CD11b (M1/70; TONBO Biosciences), FITC-conjugated anti-CD31 (#390; Thermo Fischer Scientific), FITC-conjugated anti-CD45 (30-F11; TONBO Biosciences), and FITC-conjugated anti-TER119 (35–5921; TONBO Biosciences). For fluorescence activated cell sorting (FACS) of human myoprogenitor/stem cells, dissociated tissues were washed twice with PBS(-) and incubated with APC-conjugated anti-CD29 (TS2/16; BioLegend), PE-conjugated anti-ITGA7 (3C12; MBL), FITC-conjugated anti-CD11b (M1/70; TONBO Biosciences), FITC-conjugated anti-CD31 (Wm59; BioLegend), FITC-conjugated anti-CD45 (2D1; BioLegend), and FITC-conjugated anti-TER119 (35–5921; TONBO Biosciences). As a negative control, cells were treated with APC-, PE-, or FITC-conjugated isotype IgGs. Detailed information for the antibodies were declared in Table S1. The CD11b/CD31/D45/TER119^negative^ and ITGA7/CD29 ^positive^ population was sorted as a muscular progenitor cell fraction (hereafter referred to as MPCs) including satellite cells using FACS Aria II (BD Biosciences). Details of the antibodies used in this study were declared in Table S3.

### Cell culture of mouse and human myoblasts

Tissue digestion was performed described above. Filtered cells were centrifuged at 1500 rpm for 10 min, and the resultant pellet was resuspended in 10% FCS-DMEM. The cells were then seeded in 10-cm dishes, and the medium was changed after 12 h to remove debris and suspended cells. Cells that had grown on the dish for 72 h were used as primary cells. For human tissues, collagenase treatment, filtration, and seeding into 10-cm dishes were performed as above, and cells that emerged in 1 week were used as the primary cells. The attached and expanded myoblasts were collected by enzymatic treatment using 0.25% trypsin–0.02% EDTA, harvested in 12-well plates at 2 × 10^5^ cell/well, and used for the experiments within 24 h. Passages were not applied to either cell line. The attached and expanded myoblast cells were collected by enzymatic treatment using 0.25% Trypsin–0.02% EDTA, harvested into 12-well plates, and used for the experiments.

### miRNA array

Total RNA was collected from the exosomes purified as described above using TRI Reagent (Molecular Research Center, Inc). After DNase treatment, the RNA was labeled using a FlashTag Biotin HSR labeling kit (Affymetrix) according to the manufacturer’s instructions. The resultant mix of each sample was applied to an array on the Affymetrix GeneChip miRNA 4.0 Arrays (Affymetrix). Next, the probe arrays were washed, stained, scanned, and analyzed using the Affymetrix GeneChip Fluidics Station 450.

### qRT-PCR for miRNA

Total RNA was collected from MSCs using TRI Reagent (Molecular Research Center, Inc) and reverse-transcribed using an miScript II RT Kit (Qiagen). Quantitative PCR was performed using an miScript SYBR Green PCR Kit and miScript Primer assay for *mmu-Let-7d-3p*, *hsa-Let-7d-3p*, and *has/mmu-U6 snRNA* (Qiagen). PCR amplifications were performed on a CFX Connect Real Time PCR System (Bio-Rad Laboratories) following the manufacturer’s instructions. To quantify the relative expression of miRNA, the Ct (threshold cycle) value was normalized to that of *U6 snRNA* and compared with a calibrator using the ΔΔCt method (ΔΔCt = ΔCt _sample_ – ΔCt _control_). Data are expressed as the mean value ± SD of six animals in *in vivo* experiments and three replicates in *in vitro* experiments. Statistical significance was evaluated using Student’s *t* test with the JMP software version 10.0.0 (SAS Institute).

### qRT-PCR for mRNA

Total RNA was collected from the tissues, MPCs were sorted by FACS or primary cultures, and reverse transcription was performed using a PrimeScript RT Master Mix Kit (TaKaRa Bio Inc). Quantitative PCR of total cDNA was performed using Perfect real-time SYBR Green II (TaKaRa Bio). PCR amplification was performed on a Thermal Cycler Dice Real Time System Single at 95 °C for 20 s, followed by 40 cycles at 95 °C for 5 s and 60 °C for 30 s. To prevent contamination of genomic DNA, we designed all primers to span at least one intron. Data are expressed as the mean value ± SD of six animals in *in vivo* experiments and three replicates in *in vitro* experiments. Statistical significance was evaluated using Student’s *t* test with the JMP software, version 10.0.0 (SAS Institute). Primer sequences are listed in Table S4.

### Target prediction of *Let-7d-3p*

To identify the target of *Let-7d-3p*, which can regulate antioxidant genes, we performed a target search using miRDB (http://mirdb.org/) and TargetScan v8.0 (http://www.targetscan.org/vert_80/). *In silico* ChIPseq analysis was performed using ChIP-Atlas (DBCLS and Kyusyu University) and Integrative Genomics Viewer Ver2.3.89 (Broad Institute) using a Gene Expression Omnibus dataset.

### Treatment of primary myoblast cells with mimic RNA of Let-7d-3p, exosomes, siRNA, cytokines, or NFκB inhibitors

Primary myoblast cells were transfected with mimic RNA of *mmu/hsa-Let-7d-3p* (Ajinomoto Bio-Pharma Services, GeneDesign, Inc) or scrambled RNA using Lipofectamine RNAiMAX (Thermo Fisher Scientific) following the manufacturer’s instructions. The sequence of mature *Let-7d-3p* is completely identical between humans and mice; thus, we used the same miRNA mimic sequence: 5′-CUAUACGACCUGCUGCCUUUCU-3′. For the RNA interference experiment, 10 nM siRNA (Table S5) was transfected into the primary myoblast cells using Lipofectamine RNAiMAX following the manufacturer’s instructions.

For the treatment with exosome to the primary cultures, the amount of exosomes added to the cell culture was normalized to the protein concentration. The myoblast cells were supplemented with 10 pg exosomes and analyzed after 48 h. IL-1β and TNF-α were added to myoblast cells at 1 ng/ml for 24 h. NF-κB activity was inhibited by treatment with the benzoxathiole compound BOT-64 (Abcam) or SC-514 (Abcam) at 5 μg/ml.

### Western blotting analysis

Cellular specimens from each experiment were homogenized in SDS buffer and centrifuged at 9000*g* for 10 min at 4 °C to remove debris. Aliquots were subjected to polyacrylamide gel electrophoresis, followed by electrotransfer onto a PVDF membrane (Hybond-P; GE Healthcare Japan). The membranes were blocked overnight with Block Ace (Dainippon Sumitomo Pharma) and then probed with primary antibodies (Table S2) overnight at 4 °C. Detection was performed with horseradish peroxidase–conjugated secondary antibodies and Immunostar LD (Wako) detection reagents.

### Statistical analysis

Significant differences were detected by Tukey–Kramer honestly significant difference test or Student’s *t* test, as appropriate. Statistical significance was set at *p* < 0.05.

## Data availability

All raw data used to generate the data figures are available upon request from Dr Takeshi Teramura.

## Supporting information

This article contains supporting information (48).

## Conflicts of interests

The authors declare that they have no conflicts of interest with the contents of this article.

## Supporting information

Supplementary_information_R2 scale
