# Supplementary material for: Adipose-derived exosomes block muscular stem cell proliferation in aged mouse by delivering miRNA Let-7d-3p that targets transcription factor HMGA2
Source: J Biol Chem. 2022 Jun 6;298(7):102098. doi: 10.1016/j.jbc.2022.102098 (PMC9257422; doi:10.1016/j.jbc.2022.102098)

Supplementary Table 1. List of miRNAs showing more than 2-fold upregulation in the exosome of aged-PMAT compared with that in young-PMAT.

miRNA	Aged, avg (log2)	Young, avg (log2)	Fold Change	P-value
mmu-miR-8113	6.94	3.62	9.96	2.95E-05
mmu-miR-6239	4	1.32	6.42	0.0001
mmu-miR-7075-5p	5.71	3.37	5.04	0.0078
mmu-let-7d-3p	6.39	4.15	4.72	0.0028
mmu-miR-7653-5p	8.02	5.81	4.64	0.0018
mmu-miR-7052-5p	9.69	7.54	4.46	0.0006
mmu-miR-365-2-5p	4.09	2.38	3.27	0.0397
mmu-miR-1940	8.08	6.39	3.23	0.0013
mmu-miR-6906-5p	4.74	3.1	3.12	0.0053
mmu-miR-6898-5p	7.21	5.58	3.09	0.0004
mmu-miR-677-3p	10.6	8.98	3.07	0.0129
mmu-miR-7040-5p	8.04	6.55	2.81	0.0008
mmu-miR-6966-5p	6.28	4.8	2.8	0.0073
mmu-miR-709	9.97	8.51	2.75	0.0015
mmu-miR-7025-5p	6.04	4.62	2.67	0.0114
mmu-miR-1224-5p	12.61	11.24	2.59	0.0007
mmu-miR-7030-5p	6.7	5.36	2.52	0.0167
mmu-mir-6236	5.6	4.33	2.41	0.0013
mmu-miR-490-5p	3.05	1.79	2.4	0.0477
mmu-miR-92a-3p	9.48	8.22	2.38	0.0035
mmu-miR-3544-3p	8.31	7.08	2.35	0.0425
mmu-miR-7016-5p	6.5	5.28	2.33	0.0311
mmu-mir-7032	3.2	1.99	2.31	0.0384
mmu-miR-101a-5p	5.81	4.63	2.27	0.0382
mmu-miR-3093-3p	6.45	5.27	2.26	0.0052
mmu-miR-30c-5p	9.29	8.14	2.22	0.0083
mmu-miR-7076-5p	5.91	4.78	2.19	0.0393
mmu-miR-3104-5p	7.76	6.69	2.1	0.0101

Supplementary Table 2. Target sequence of mmu-Let7d-3p and hsa-Let-7d-3p to the Hmga2/HMGA2 gene. Prediction was performed using TargetScan.

Predicted target region and miRNA		Sequence	Context++ score percentile
Position of Hmga2 3' UTR	5'	...GUGACCGGGGGCAUUCGUAUAA...	
		| | | | | |	-0.51
mmu-Let-7d-3p	3'	UCUUUCCGUCGUCCAGCAUAUC	
hsa-Let-7d-3p	3'	UCUUUCCGUCGUCCAGCAUAUC	-0.59

Supplementary Table 3. Antibodies used in FACS and WB analyses in the present study.

Application	Antibody (Clone, Cat No)	Company	Reactivity	Dilution
FACS	CD11b (M1/ 70, 35-0112)	TONBO	Ms / Hm	1 μg / 10^7 cells
	CD29 (TS2/16, 303008)	Biolegend	Hm	1 μg / 10^7 cells
	CD29 (HMβ1-1, 102216)	Biolegend	Ms	1 μg / 10^7 cells
	CD31 (WM59, 303104)	Biolegend	Hm	1 μg / 10^7 cells
	CD31 (390, 11-0311)	Thermo Fisher Scientific	Ms	1 μg / 10^7 cells
	CD34 (MEC14.7, 119321)	Biolegend	Ms	1 μg / 10^7 cells
	CD45 (2D1, 368508)	Biolegend	Hm	1 μg / 10^7 cells
	CD45 (30-F11, 35-0451)	TONBO	Ms	1 μg / 10^7 cells
	CXCR4 (L276F12, 146517)	Biolegend	Ms	1 μg / 10^7 cells
	Itga7 (3C12, K0046-5)	MBL	Ms / Hm	10 μl / 10^7 cells
	TER-119 (TER-119, 35-5921)	TONBO	Ms	1μg / 10^7 cells
WB	Gapdh (5A12, 014-25524)	FUJIFILM	Ms / Hm	1 / 5,000 in Immuno-enhancer
	Hmga2 (D1A7, #8179)	Cell Signaling	Ms / Hm	1 / 1,000 in Immuno-enhancer
	NF-κB p65 (D14E12, #8242)	Cell Signaling	Ms	1 / 1,000 in Immuno-enhancer
	Phospho-NF-κB p65 Ser536 (93H1, #3033)	Cell Signaling	Ms	1 / 1,000 in Immuno-enhancer

Supplementary Table 4. Primer sequences used in qRT-PCR analysis.

Species	Gene	Forward	Reverse
**Mouse**	*Ccna1*	ATGAGCAGTACAGGAGGACC	AGAGAGCACTTTCTTTCCAGC
	Ccnb1	AGAGCTACAGGCAAGAGTGC	ACACAGGCACCTTCTCTACAG
	Cdk6	TGCTGAGGCACCTGGAGACC	TAGTGTAAGCTTGGTTTCTCTG
	Hmga2	TGCCACAGAAGCGAGGACG	TCTTCCTCTGGGTCTCTTAG
	Id1	TGAACGGCGAGATCAGTGC	TCAGCGACACAAGATGCGATC
	Id2	TGAACACGGACATCAGCATCC	AGCCACAGAGTACTTTGCTATC
	Lin28a	TCGGTGTCCAACCAGCAG	ACGTTGAACCACTTACAGATGC
	Lin28b	TCATCTCCATGATAAGTCGAGAG	TCAAGCTTCTAAATCCTTCCATG
	Notch1	TGGACAAGATCAATGAGTTC	ACACTCATCCACATCATACTG
	Pax3	AGGTACCAGGAGACAGGCTC	TGAGCAATTTGTCTCTGATTTC
	Pax7	AAAGCCAAACACAGCATC	TCGGGTTCTGATTCCAC
**Human**	*CCNA1*	TATCCAGTCTTGGCACAGATG	TCTGGCTGCTTCTTCATGTAG
	CCNB1	AGCTGATCCAAACCTTTGTAG	TCTCATGTTTCCAGTGACTTCC
	CDK6	ACTTGGATAAAGTTCCAGAGC	ATGAAGAAAGTCCAGACCTC
	HMGA2	AAGCAGAAGCCACTGGAG	TGAGCAGGCTTCTTCTGAAC
	ID1	ATCGACTACATCAGGGACC	TCGGATCTGGATCTCACCTC
	ID2	ACCACCCTCAACACGGATATC	TCAGCCACACAGTGCTTTGCTG
	NOTCH1	AGGAAACAACTGCAAGAACG	TGGCACTCGTCCACATCCTC
	PAX3	AGCATCGACGGCATCCTGAG	AGTGGTAAATCTGGTTCAGAGTC
	PAX7	TACCAGGAGACCGGGTCCATC	TTCCCTCTTGTACTCCTCAATC

Supplementary Table 5. siRNA sequences used in this study.

Target gene	Sense	Antisense
Mouse Hmga2	GGA AAU GGC CAC AAC AAG UdTdT	ACU UGU UGU GGC CAU UUC CdTdA
Scrambled sequence	GUA CUC AUG CUA UAU UGC UdTdT	AGC AAU AUA GCA UGA GUA CdTdT

Supplementary figure 1

Expression of stemness- and cell cycle-associated genes in young or aged MPCs sorted as the CD11b/CD31/D45/TER119^{negative} and ITGA7/ CD29 ^{positive} population.

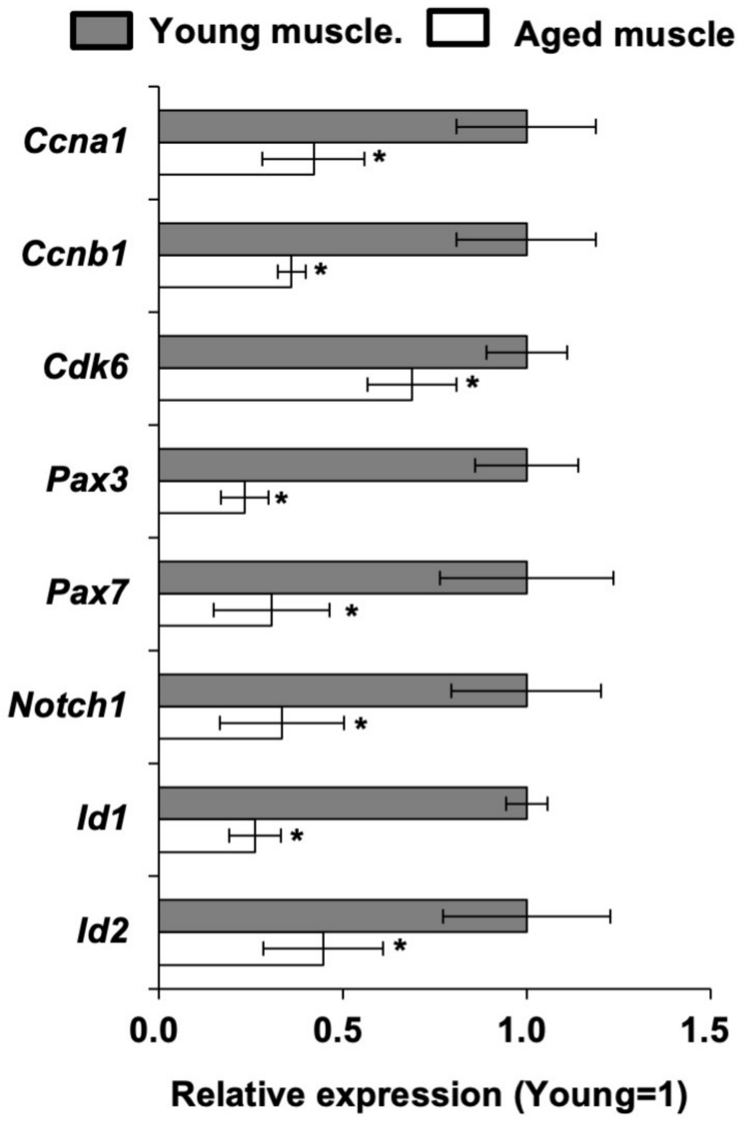

Supplementary figure 2

FACS analysis of mice primary MPCs. Primary cells prepared from mice muscular tissues contained CD29/Itga7^{positive} progenitor cells at approximately 84%. Pax7-expressing putative stem cells were included in the CD29/Itga7^{positive} population at approximately 34%.

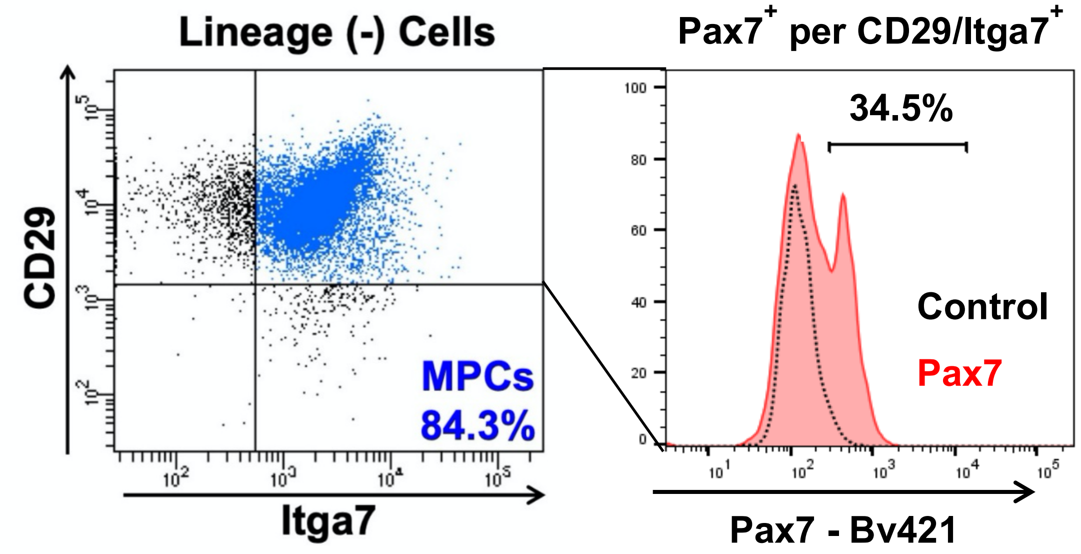

Supplementary figure 3

Evaluation of isolated exosome. (A) Morphological integrity of the isolated exosome was observed by transmission electron microscopy. (B) Correction of exosome was determined by western blotting analysis for the exosome markers CD9, CD63, CD81, and Tsg101.

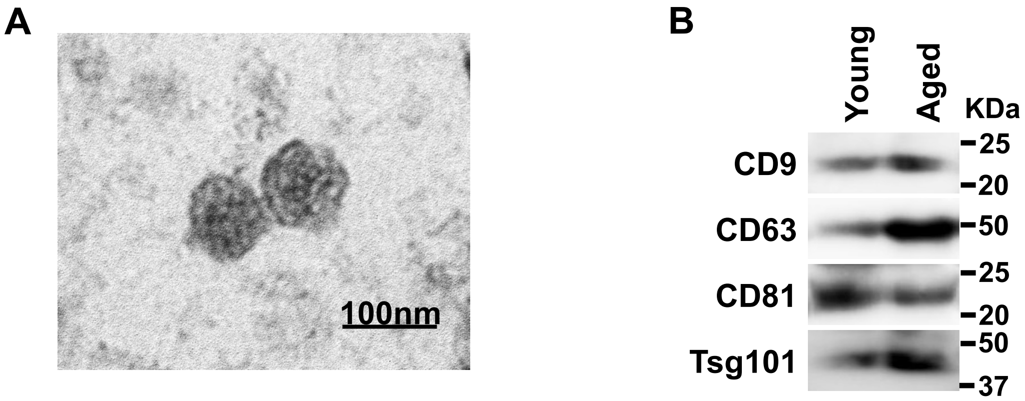

Supplementary figure 4

Effect of *Let-7d-3p* mimic transfection on cell proliferation and gene expression in the HMGA2-overexpressing myoblast cells. (A) Overexpression of HMGA2 was confirmed by western blot analysis (a). The overexpressed Hmga2 cDNA that lacks 3'UTR was not repressed by the *Let-7d-3p* mimic RNA (b). (B) *Let-7d-3p* did not change the proliferation and (C) gene expression of self-renewal associated genes under the HMGA2 overexpression condition, indicating that the suppression of cell proliferation/self-renewal by *Let-7d-3p* was mediated by the downregulation of HMGA2. For the HMGA2 overexpression, a piggyBac transposon-based system (pPB) was used. Mouse Hmga2 cDNA was amplified with Tks Gflex DNA polymerase from the mouse myoblast cDNA. Then the PCR amplicons were digested with EcoRI and ligated into the EcoRI sites of pPB-CAG-IRES-Puro plasmid (Takehara et al., Scientific Reports 2015, 30;5:14722). The pPB-CAG-HMGA2-IRES-Puro plasmid was co-transfected with pCAG-PBase plasmid to mouse primary MPCs using Lipofectamine 3000 (Thermo Fisher Scientific). To prepare the control cells, pPB-CAG-puro plasmid and pCAG-PBase plasmid were introduced into mouse primary MPCs. After 48 h of transfection, the MPCs were treated with 1 μg/mL puromycin (Thermo Fisher Scientific) for 72 h, and expanded cells were used for the experiment.

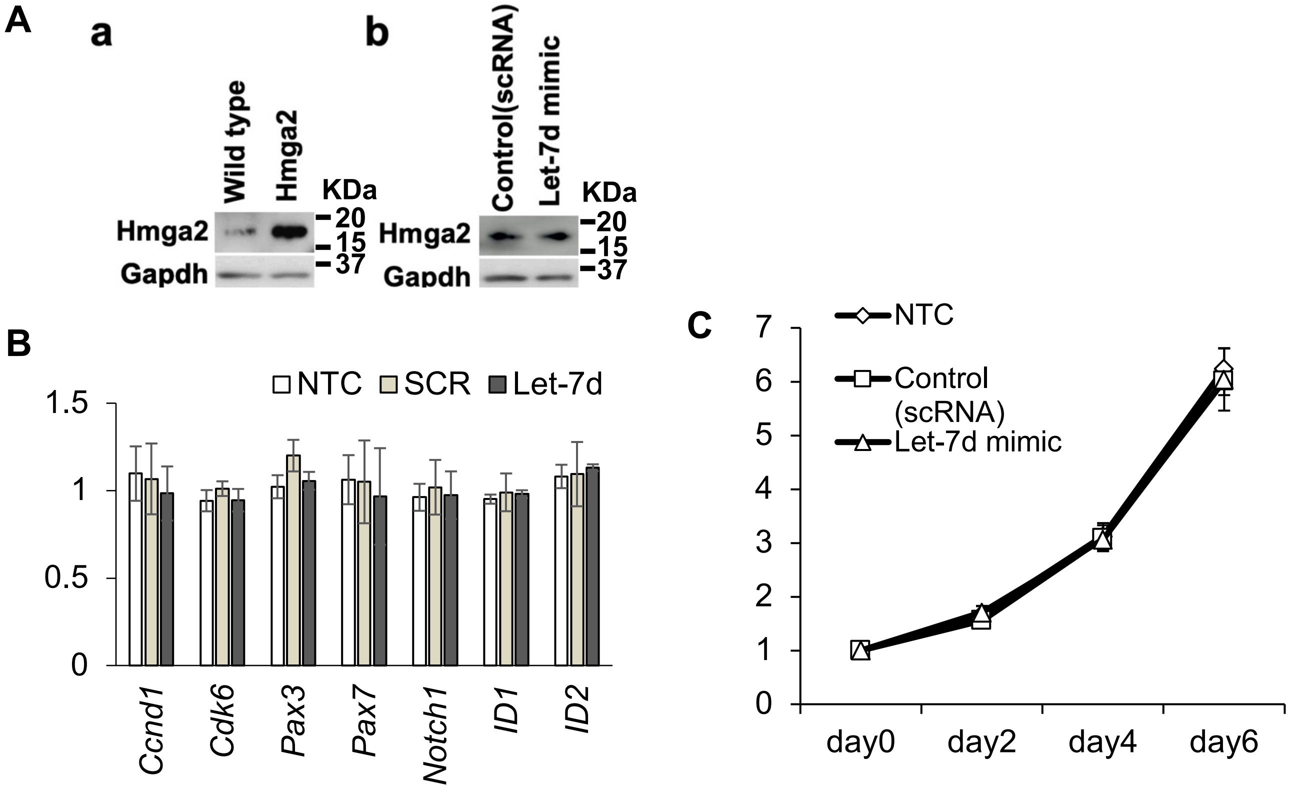

Supplementary figure 5

Western blot-based confirmation of the effect of NF-κB inhibitors.

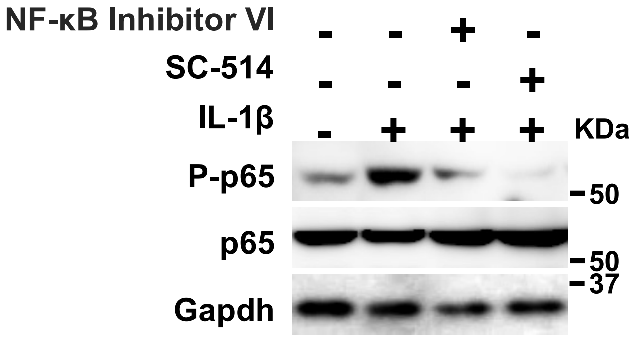

Supplementary figure 6

Changes in the expression of myogenic marker genes by the transfection of *Let-3d-3p* miRNA mimic in mice and human primary MPCs.

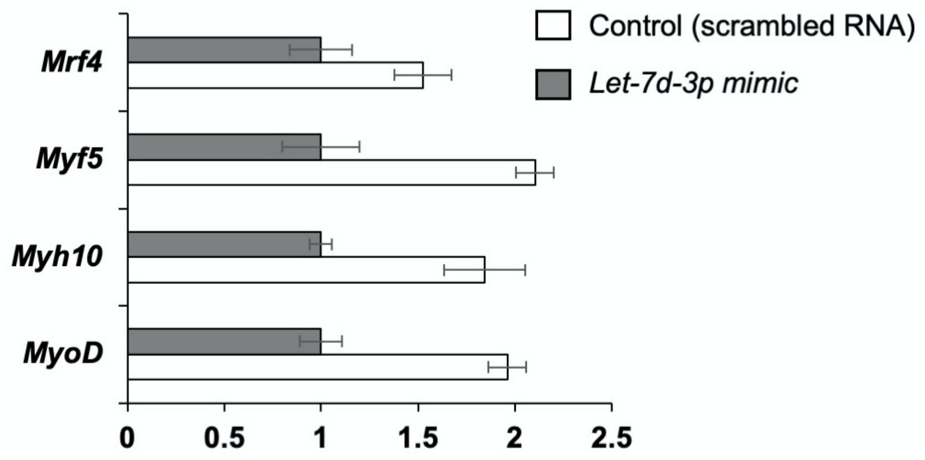

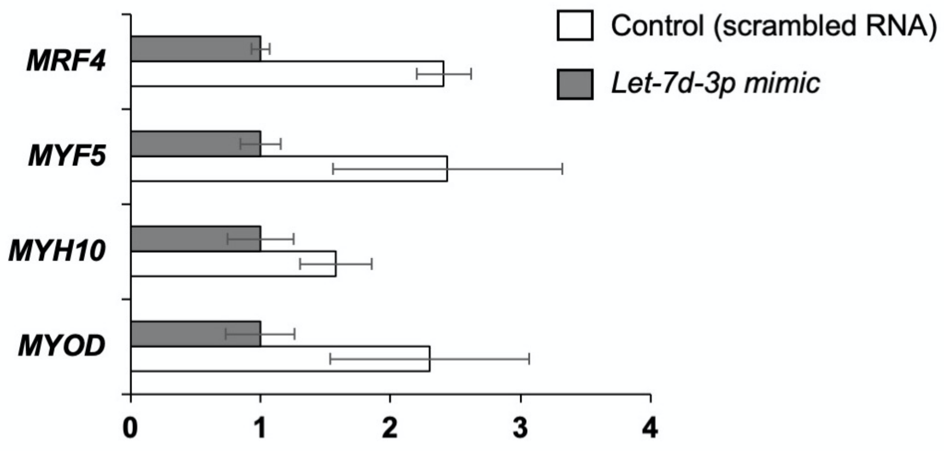

Supplement: Supplementary_information_R2 scale [file mmc1.pdf]
